# The knowledge domain of cognitive neuroscience of aging: A Scientometric and bibliometric analysis

**DOI:** 10.3389/fnagi.2023.999594

**Published:** 2023-02-09

**Authors:** Jiaxing Jiang, Lin Fan, Jia Liu

**Affiliations:** ^1^Research Institute of Foreign Language, Beijing Foreign Studies University, Haidian, Beijing, China; ^2^National Research Center for Foreign Language Education, Beijing Foreign Studies University, Haidian, Beijing, China; ^3^School of Foreign Studies, Hebei Normal University, Shijiazhuang, China

**Keywords:** cognitive neuroscience of aging, bibliometric analysis, cognitive aging, scientometric analysis, CiteSpace

## Abstract

Cognitive neuroscience of aging (CNA) is a relatively young field compared with other branches of cognitive aging (CA). From the beginning of this century, scholars in CNA have contributed many valuable research to explain the cognitive ability decline in aging brains in terms of functional changes, neuromechanism, and neurodegenerative diseases. However, very few studies have systematically reviewed the research in the domain of CAN, with regard to its primary research topics, theories, findings, and future development. Therefore, this study used CiteSpace to conduct a bibliometric analysis of 1,462 published articles in CNA from Web of Science (WOS) and investigated the highly influential and potential research topics and theories of CNA, as well as important brain areas involved in CAN during 2000–2021. The results revealed that: (1) the research topics of “memory” and “attention” have been the focus of most studies, progressing into a fMRI-oriented stage; (2) the scaffolding theory and hemispheric asymmetry reduction in older adults model hold a key status in CNA, characterizing aging as a dynamic process and presenting compensatory relationships between different brain areas; and (3) age-related changes always occur in temporal (especially the hippocampus), parietal, and frontal lobes and the cognitive declines establish the compensation relationship between the anterior and posterior regions.

## 1. Introduction

Although the field of cognitive neuroscience of aging (CNA) has existed for decades, it has not been considered as a specific discipline until recently, because the cognitive and neural mechanisms of age-related changes in cognition used to be studied independently of each other (Cabeza et al., 2018). As people age, many neurological diseases (e.g., Parkinson’s disease, Alzheimer’s disease, and language disorders) and cognitive declines (e.g., hypomnesia) adversely affect the brain. In cognitive psychology of aging, behavioral studies have investigated the effects of aging on memory (Noice and Noice, 2006; Gazzaley et al., 2007), attention (Lawo and Koch, 2014; Golob and Mock, 2018), and the relationship between aging and individual differences, such as distraction control (Darowski et al., 2008), social status and beliefs about cognitive aging (Weiss and Weiss, 2016). Studies of cognitive aging in neuroscience, in parallel, probed into the effects of aging on the anatomy and physiology of the brain (Cabeza et al., 2018), with the help of different techniques in neuroscience such as functional magnetic resonance imaging (fMRI), event-related potentials (ERPs), diffusion tensor imaging (DTI), magnetoencephalogram (MEG), and positron emission tomography (PET).

During an individual’s lifespan, several structural and functional physiological changes will occur as he/she ages (Drag and Bieliauskas, 2010). The frontal cortex is most affected structurally when the aging brain starts to decline and it will decline the fastest in comparison to the temporal, parietal, and occipital cortices (Haug, 1983; Haug and Eggers, 1991). Age-related structural changes are also found in the anterior region (Head et al., 2004; Pfefferbaum and Sullivan, 2005), hippocampus (Morrison and Hof, 1997; Raz et al., 1998; Raz, 2005; Head et al., 2008), and cerebrovascular region (Gazzaley and D’Esposito, 2005). In neuroimaging studies, Cabeza (2002) found that prefrontal activation during cognitive processing tended to be less lateralized in old adults than in young adults and thus proposed the hemispheric asymmetry reduction in older adults (HAROLD) model and Davis et al. (2008) found a posterior–anterior shift and explained such a shift across diverse cognitive functions. Aside from studies on the structural and functional changes in aging brains, published articles also concerned CA theories such as functional compensation theory (Cabeza et al., 2004), scaffolding theory (Park and Reuter-Lorenz, 2009), and the theory of dedifferentiation (Li and Lindenberger, 1999; Li and Sikström, 2002).

The above-mentioned achievements have been reviewed by a series of academic articles on cognitive aging studies. Those review articles cover wide-ranging topics of CA studies, including cognitive abilities (Reynolds and Finkel, 2015), psychosocial protective factors (Zahodne, 2021), environment support (Lindenberger and Mayr, 2014), genetic biomarkers (Lin et al., 2017), neural correlates of cognitive reserve *via* fMRI (Anthony and Lin, 2018), and the development history of CA (Drag and Bieliauskas, 2010; Anderson and Craik, 2017). There is also a bibliometric analysis of CA studies that critically evaluated common theories of age-related cognitive decline (Ebaid and Crewther, 2020), and a meta-analysis assessed the aging effects in attention-related tasks (Vallesi et al., 2021; Mckay et al., 2022). Nevertheless, these published reviews combined the findings of CNA and cognitive psychology of aging. As the CNA research grows, a group of terms (e.g., reserve, maintenance, compensation) have been loaned to describe the qualitative and quantitative differences in the brain structure and function to advance current theories of brain aging and cognitive declines (Cabeza et al., 2018). Under this circumstance, these studies may construct their own academic relations, which cannot be clearly revealed in the review of *CA.* In addition, the existing reviews mostly focused on an individual factor or theory development in CA without revealing the general disciplinary development and findings in CNA. Therefore, the main research topics, theories, and neuromechanisms in CNA are not clear at present. For instance, research topics like memory, attention, perception, language, feelings are all popular in cognitive psychology and neuroscience studies. However, whether the researchers in the field of CNA have also investigated those topics or they concern only one or two of the topics and to what extent those popular topics have been studied are not very clear. In the research of those popular topics, what are the most influential theories that have been accepted by scholars in CNA is unclear likewise. In addition, those studies have probed into many brain areas concerning different cognitive abilities (especially of Alzheimer’s and Parkinson’s patients), but whether there exists important consensus about the relationship between those brain areas remains obscure. It apparently depends on the analysis of the abundant literature of CNA to answer these disciplinary fuzzy points. Consequently, this study adopted a bibliometric method to delve into the CNA literature and sorted out the development of CNA over the past two decades.

Unlike previously published bibliometric analyses of CA (e.g., Othman et al., 2022) that focused on the productivity authors/countries and collaboration, the current study focused on the topics and theories in the field of CNA and generalized the above research gaps into three questions: (1) What are the crucial research topics (i.e., objectives or psychological phenomena) and to what extent have they developed in the domain of CNA? (2) What are the influential and potential theory (or theories) that will lead to transformative discoveries in CNA? and (3) What are the age-related physiological changes in particular brain area (s) relating to the research topics?

According to Paul and Rialp (2020), there are three types of systematic literature reviews: (1) the domain-based review, which synthesizes research into various themes; (2) theory-based review, which concentrates on how research from the same body of literature applies theories; and (3) the method-based analysis, which focuses on the methodologies used in a body of literature. In the current research, the domain-based review is a reasonable choice because it combines the bibliometric analysis and content analysis. The current bibliometric techniques have been applied quantitatively to evaluate the research trends and tacit scientific knowledge in published academic literature *via* mathematical, statistical, and other measurement methods (Yu et al., 2017). The bibliometric approach can “help analysts visualize and break down co-citation networks on the basis of the algorithm of co-citation matrix” (Fu et al., 2020, p: 2) and provide both a diverse range of relevant topics in a particular research field and the knowledge flow between topics (Chen et al., 2014). Among the various software available for performing bibliometric analysis, CiteSpace is employed widely to detect the emerging trends, knowledge foundations, and innovations in academic fields (Xu and Yu, 2019). Not only does it provide the basic co-citation analysis, but it also provides the betweenness centrality (BC) and citation burst of co-citation and co-word analyses (Chen, 2016). The results of these two statistical analyses can foretell the development of a particular field (Chen, 2017).

Considering the three research questions that required the visualization of knowledge domain and BC values to tell the diachronic changes in CNA, this study adopted CiteSpace (v.6.1.R2) for data analysis. Our study synthesized networks of co-citation references based on bibliographic records retrieved from Web of Science (WOS), published from 2000 to 2021. The following sections started with a brief introduction of our research method, including the delineation of the essential thresholds in CiteSpace, followed by a depiction of CNA studies in terms of cluster analysis (based on co-citation analysis) and keyword analysis (based on co-word analysis). Finally, the three research questions were addressed based on the discussion of our collected data and conclusions were drawn.

## 2. Research method

A bibliometric analysis is a computer assisted review for identifying core research or authors, and their relationships by examining publications related to a specific topic or field (DeBellis, 2009). WOS and Scopus have been the two most widely used databases for bibliometric analysis (Singh et al., 2021). Compared with Scopus, WOS is more rigorous and about 99.11% of the journals indexed in WOS are also indexed in Scopus (Singh et al., 2021). Therefore, the data for this research were retrieved and downloaded from WOS database, including Science Citation Index Expanded (SCIE), Social Science Citation Index (SSCI), Arts and Humanities Citation Index (A&HCI), Conference Proceedings Citation Index-Science (CPCI-S), Conference Proceedings Citation Index-Social Science & Humanities (CPCI-SSH), and Emerging Sources Citation Index (ESCI).^1^

### 2.1. Data collection

**Figure 1:**
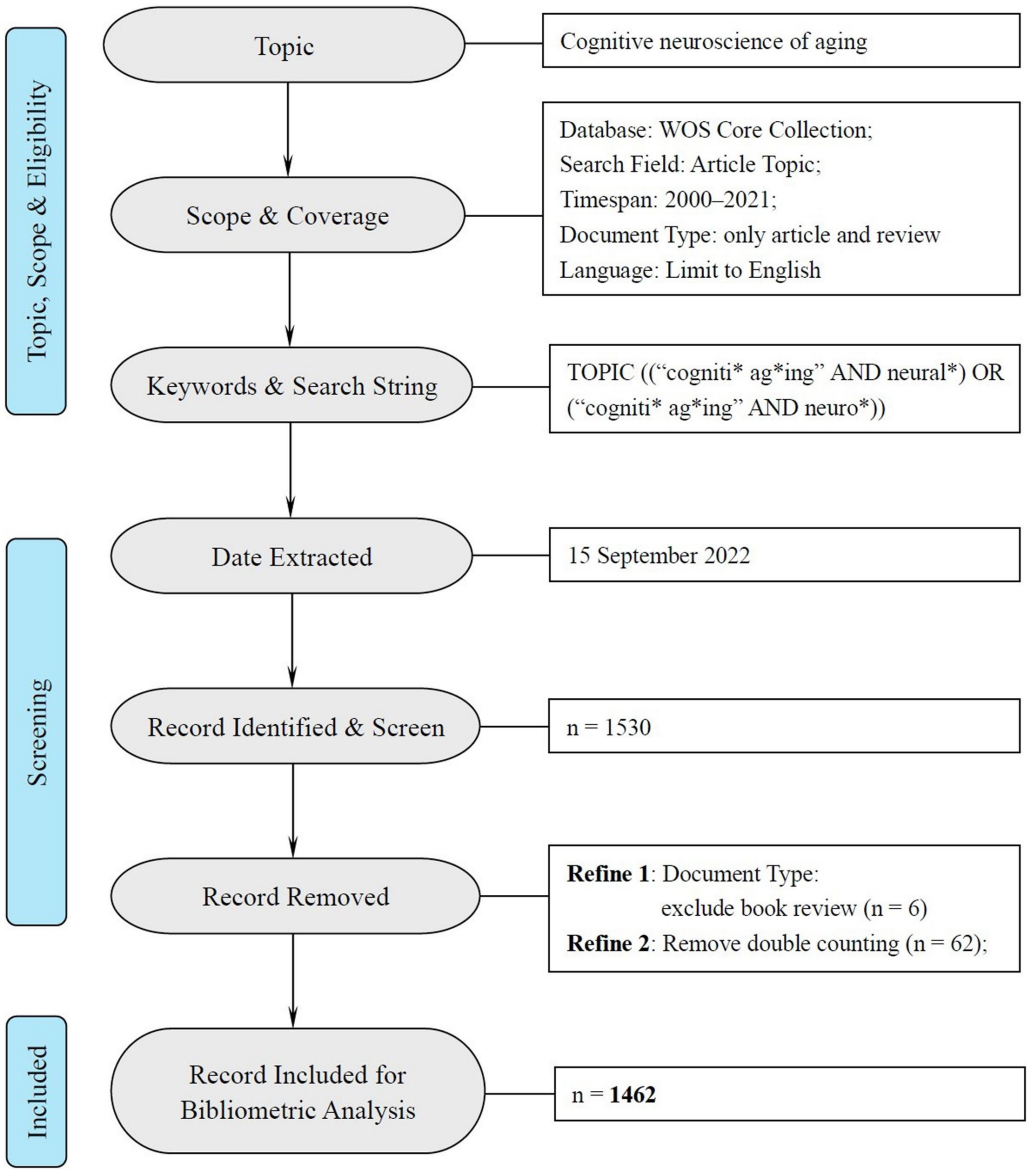
PRISMA flow chart of data inclusion and exclusion.

Following the bibliometric analysis of cognitive aging by Othman et al. (2022), this research adopted their search term “cogniti*” and “ag*ing” with another two additional terms “neural*” and “neuro*” to define the time span in the field of neuroscience (see Figure 1). The present study included all the research articles and reviews during 2000 to 2021 written in English.^2^ The data was retrieved through “TOPIC” algorithm (including the title, abstract, and keywords in each article) in WOS. To guarantee the retrieval ratio, we adopted the “remove duplicate (WOS)” function in CiteSpace to avoid double counting. Finally, 1,462 documents were identified for further analysis and the data inclusion was illustrated, according to the PRISM 2020 statement for systemic review^3^ in Figure 1.

### 2.2. Thresholds in CiteSpace

**Figure 2:**
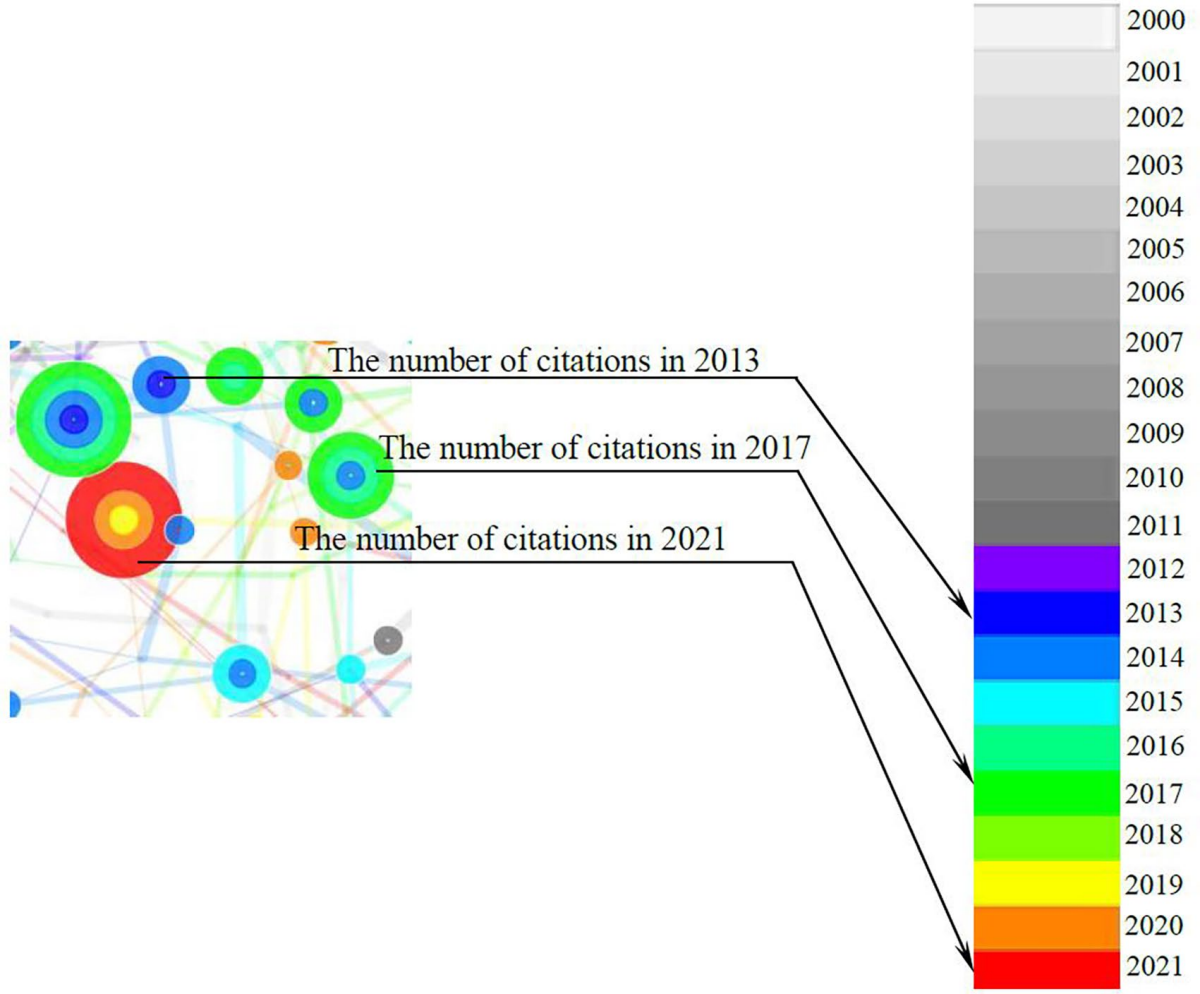
An example of nodes.

CiteSpace is designed to synthesize and visualize the literature in the form of a co-citation network, with co-cited references visualized as nodes, as shown in Figure 2. The node’s citation timespan is denoted by the different colors, from nearly white (i.e., year 2000) to red (i.e., year 2021). The thicker the node’s tree ring, the more frequently the reference is cited. According to Chen (2006), the node can be further categorized into various cultures based on the relativity and interconnectivity among references on a specific topic. The essential thresholds employed in this study are listed in Table 1.

**Table 1:** Essential thresholds of the co-citation and co-word analyses in CiteSpace.

Apparatus	Threshold	Definition
Modularity (Q)	> 0.3	It refers to whether the co-citation clusters in the given field are clearly defined (Chen et al., 2010; Chen, 2016).
Silhouette	> 0.5	It refers to whether the clustering effects are reasonable, and whether all clusters are well connected with one another (Chen et al., 2010; Chen, 2016)
Betweenness centrality (BC)	> 0.1	BC is a structural metric for qualifying the academic impact of a reference in citation networks (Li and Chen, 2016). A high value of BC usually leads to transformative discoveries (Chen et al., 2009; Chen, 2017).
LLR		It is a log-likelihood ratio test to effectively recognize labels within a cluster (Chen et al., 2010; Chen, 2012).

The calculation was performed with the following settings: g-index = 25, TopN = 50, TopN% = 10%, and time slice = 1. The values of modularity and silhouette of all clusters reached the threshold; thus, other algorithms (e.g., pathfinder, pruning sliced networks, and pruning the emerged network) were not manipulated in generating clusters. These apparatuses were identical in performing co-citation and co-word analyses and were derived empirically (see Chen and Song, 2019).

## 3. Results

**Figure 3:**
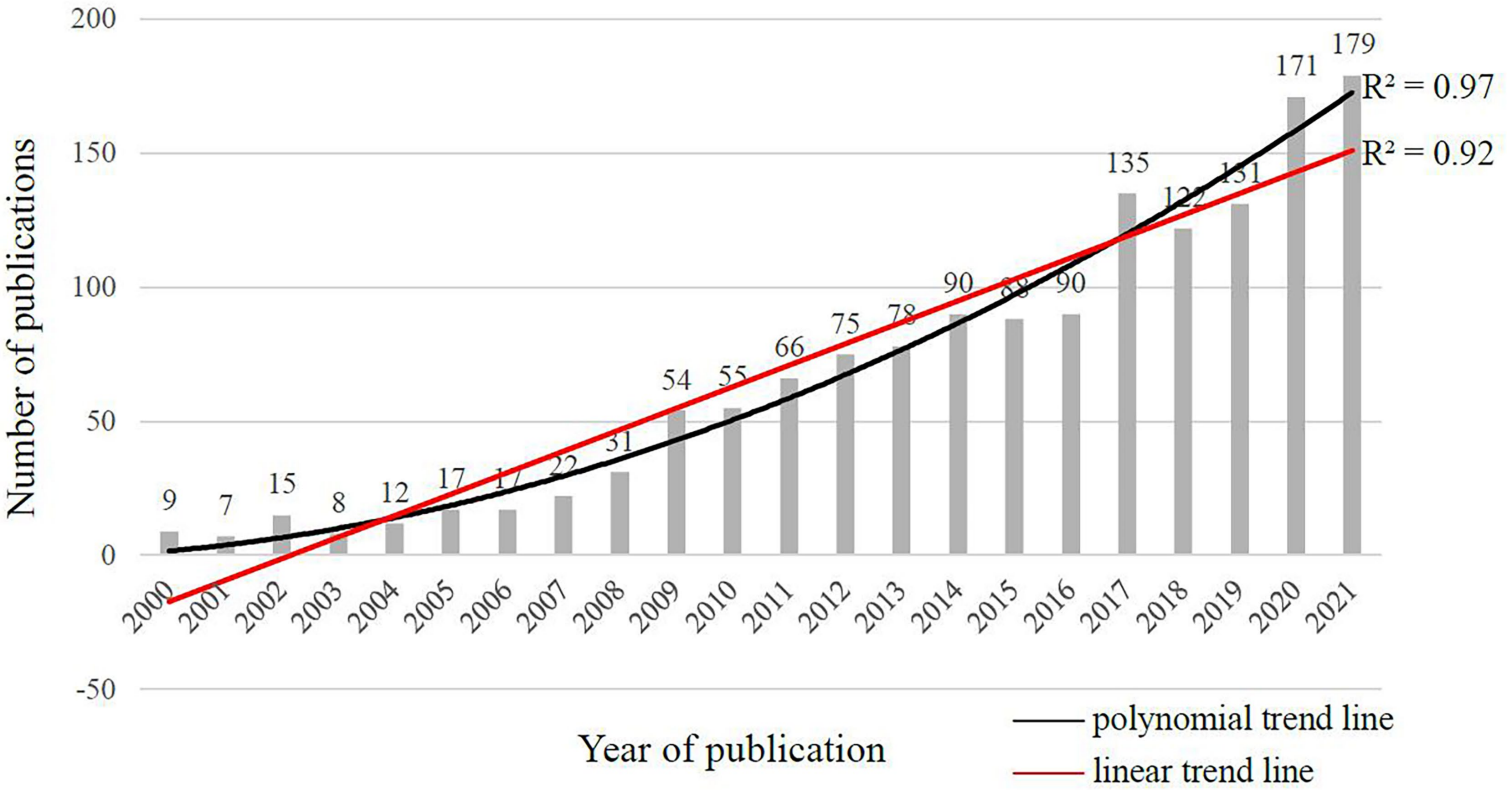
Annual publication counts of CNA from 2000 to 2021 in the web of science core collection based on the search topics.

Figure 3 displayed the annual number of the literature of CAN studies published from 2000 to 2021. The annual publication counts confirmed Cabeza et al. (2018) statement that the CNA field had not been considered as a specific discipline until recently. The publication counts before 2009 were less than 50 articles per year and less than 100 articles per year prior to 2017.^4^ Apparently, CNA started early at the beginning of this century but has not entered a mature period, and at present, the number of academic investigations per year remains relatively insufficient. Nevertheless, both the ploynominal (R^2^ = 0.97) and linear (R^2^ = 0.92) trend lines suggested a continuous increase in the number of publications, which implied that the research of CAN had started to attract more attention, probably because of the worldwide population aging.

### 3.1. Co-citation analysis: Major topics and influential theories

**Figure 4:**
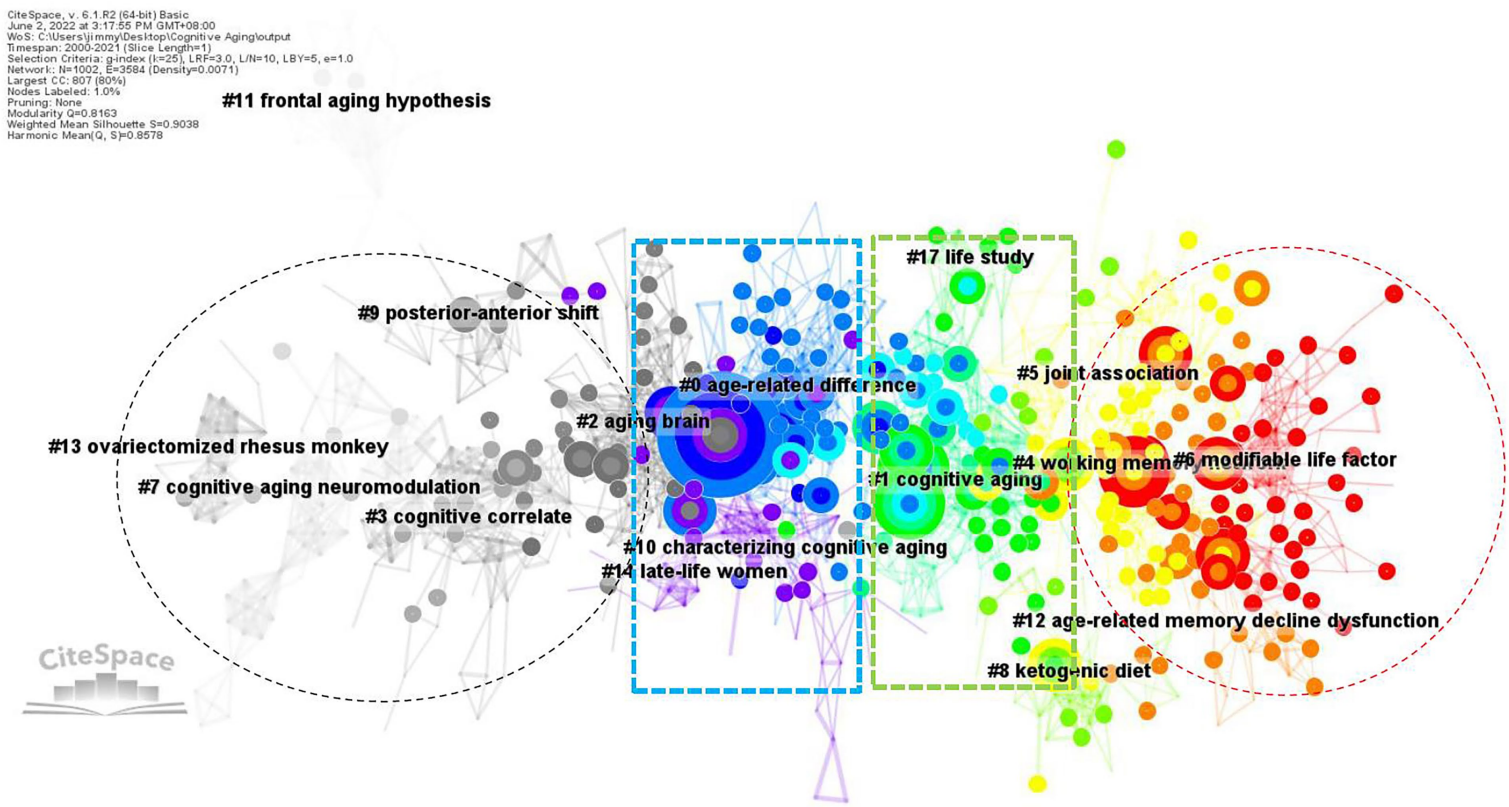
Landscape view of the co-citation network in CNA from 2000 to 2021.

The co-citation function in CiteSpace constructed networks of the cited references and established network models. These networks were divided into (co-citation) clusters of references. As shown in Figure 4, each cluster contained a theme, namely, the label shared by the cited references. The co-citation analysis visualized 16 major clusters (Modality = 0.8163 and Silhouette = 0.9038) labelled by LLR. Table 2 presented the silhouette values of each cluster from the largest cluster #0 (age-related difference) to the smallest cluster #17 (life study). It was obvious that the size of each cluster had a wide range, from 107 to 15, showing the keywords of CAN studies shared by the literature in each cluster. It clearly revealed the different status of the references in each cluster. In other words, the references in cluster 0# were theoretically more influential to CNA than those in cluster 17#.

**Table 2:** Summary of the largest 16 clusters of CNA research.

Cluster ID	Size	Silhouette	Label (LLR)	Year
0	107	0.862	age-related difference	2013
1	99	0.853	cognitive aging	2015
2	87	0.84	aging brain	2010
3	76	0.933	cognitive correlate	2006
4	63	0.902	working memory network	2019
5	59	0.857	joint association	2018
6	55	0.932	modifiable life factor	2020
7	46	0.959	cognitive aging neuromodulation	2003
8	35	0.954	ketogenic diet	2018
9	33	0.926	posterior–anterior shift	2008
10	33	0.982	characterizing cognitive aging	2011
11	33	0.971	frontal aging hypothesis	2000
12	24	0.936	age-related memory decline dysfunction	2019
13	21	0.991	ovariectomized rhesus money	2004
14	21	0.93	late-life women	2012
17	15	0.92	life study	2016

The landscape view of the co-citation of CAN literature colorfully depicted the different periods of CNA research from 2000 to 2021 (i.e., the black, blue, green, and red colors in Figure 4) and divergent topics. Synchronically, these CNA clusters were categorized into six groups.^5^ The first group contained clusters #0 and #2. Their labels generated by CiteSpace automatically did not differ from each other explicitly, but the content of each frequently cited and high BC value articles in these two clusters primarily concerned the theoretical deliberation of aging and cognition, respectively. One significant theory, namely the scaffolding theory of aging and cognition (STAC), was elucidated by Reuter-Lorenz and Park (2014) in cluster #0. They revised their STAC model proposed in 2009, and named the revised version as STAC-r model, which combined a life-span approach with a life-course approach to reveal and predict the cognitive status and rate of cognitive changes overtime. Cluster #2 on the other hand concerned the relationship between neuroplasticity and cognitive aging. Greenwook and Parasuraman (2010) found two types of plasticity in the aging process. One was neuronal plasticity related to neuron-level changes stimulated by experience and the other was cognitive plasticity associated with adaptive changes in cognition associated with the brain activity. Neural plasticity underpinned cognitive plasticity and was stimulated by cognitive plasticity. From the perspective of STAC, neuroplasticity was deemed as a compensatory response in aging brains based on the review of age-related behavioral and neural findings by Goh and Park (2009).

The second group, including clusters #1, #3, #5, #7, #9, and #11, focused on the changes in aging brains in terms of structure, function, and neural correlates. The major research contained in cluster #1 dealt with the functional connectivity decline in aging brains. Siman-Tov et al. (2017) revealed that except for the structural changes in particular brain areas, a considerable connectivity within high-order cognitive networks occurred in middle adulthood. This finding is consistent with the concept proposed by Fjell et al. (2017) that the major part of age-related reductions in executive function pertained to the micro-and macro-structural alternations in brain connectivity. In cluster #3, Fjell et al. (2009) reviewed the changes (e.g., cortical thickness and volume) occurred in aging brains across the superior, middle, and inferior frontal gyri, superior and middle temporal gyri, precuneus, parietal cortices, fusiform and lingual gyri, and the temporal–parietal junction. Besides, Chee et al. (2009) also found grey and white matter volume loss in older adults around the primary visual cortex and bilateral superior parietal cortices. Cluster #5 was associated with AD. Studies in this cluster focused on some biomarkers like β-amyloid levels (Knopman et al., 2018), vascular health (Vemuri et al., 2018), and the tau pathology originated in brainstem nuclei (Dutt et al., 2020). Clusters #7 and #9 are both relative to the neural mechanisms of older adults. Some studies investigated the neuromodulation mechanism during aging (e.g., Li and Sikström, 2002), revealing that age-related decline in dopaminergic neuromodulation reduced the fidelity of neural information. Other studies explored the relations between cognitive aging and sensory/sensorimotor aging (e.g., Li and Lindenberger, 2002), suggesting that both two types of aging generally hindered older adults’ performance with cognitive resource overlap, competition, and tradeoffs. There were also some studies focusing on the neural mechanism underlying the functional compensation in aging brains from posterior to anterior, labeled as posterior–anterior shift in aging (PASA; e.g., Davis et al., 2008; Stevens et al., 2008; Tays et al., 2008). These findings in clusters #7 and #9 were opposed to the main argumentation in cluster #11, namely frontal aging hypothesis, which stated that “functions largely dependent on frontal regions would decline in aging, while functions largely independent of frontal lobes would remain relatively spared” (Greenwood, 2000, p: 705). Due to the compensation from frontal region to occipital region, there was only weak evidence supporting the frontal aging hypothesis (Greenwood, 2000).

The third group (i.e., clusters #4 and #12) was relevant to (working) memory capacities of older adults. Cluster #4 focused on the new methodologies for enhancing the mechanisms of optimal brain function in advancing aging. For example, Abellaneda-Pérez et al. (2019) reviewed and commented on the combination between non-invasive brain stimulation and fMRI technique. Besides, other scholars concentrated on the use of transcranial direct current stimulation (tDCS) for improving older adults’ working memory performance (e.g., Nissim et al., 2019; Rosa et al., 2019). Cluster #12 delved into the aging brain from a molecular perspective. It discussed the alteration of DNA methylation pattern in old neurons (Harman and Martín, 2020), and found that the expression of *Egr 1* and *c-Fos* was associated with aging while *Arc* was more linked to cognitive outcomes for older adults (Myrum et al., 2020).

The fourth group of studies (containing clusters #6 and #8) centered around the improvement on aging brains. Studies in this group primarily focused on the factors that caused age-related cognitive decline and mechanisms underlay such a decline. The review by Duggan and Parikh (2021) presented evidence supporting a significant role of microglia within the central nervous system, and they concluded that modifiable lifestyle factors (e.g., healthy diet or exercise and cognitive engagement) brought about cognitive benefits. Similarly, in cluster #6, another important review by Krivanek et al. (2021) also presented some positive factors for promoting cognitive capacity such as regular physical activity, heart-healthy diet, smoking cessation, and the like. Among these factors, the healthy diet had also been elucidated in cluster #8. Hernandez et al. (2018) adopted a ketogenic diet (KD) as a global metabolic strategy for improving brain function, and found that KD could be optimal for enhancing large-scale network function for higher cognition.

The fifth group of research (containing clusters #10, #13, and #14) was the only one conducting nonhuman experiments. Most animal experiments in this group took rodents or primates as their participants. For instance, in cluster #10, Bizon et al. (2012) reviewed behavioral approaches of the studies on rodents’ executive function, concentrating on those assays that would contribute to studies on human aging. Briones and Darwish (2012) probed into the association between the use of vitamin D and the improvement of age-related cognitive decline in rats, and the results suggested that vitamin D supplementation modulated age-related increase for the pro-inflammatory state and amyloid burden, which mediated the aging rats’ memory impairment. Except for vitamin D, estrogen is another important factor in this group. The studies of cluster #13 chose primates as their participants. Rapp et al. (2003), for example, employed 16 ovariectomized aged rhesus monkeys to explore their spatial working memory, and concluded that ovarian hormone status broadly influenced their memory system, which supported the concept that estrogen benefited (working) memory of women. Lacreuse (2006) reviewed the evidence for nonhuman primate experiments to detect whether those evidence could benefit our understanding of the influence of estrogen in cognitive aging. The results revealed that nonhuman primate experiments provided valuable evidence for scholars to understand the hormonal actions of brain and cognition. Such an assertion has been adopted in the studies of spatiotemporal working and recognition memory impairment in aged rhesus monkeys (Shamy et al., 2011) as well as the studies of episodic memory and executive functions of late-life women (Henderson and Popat, 2011) in cluster #14.

The last group of studies (i.e., cluster #17) was related to genome wide association that connected with late onset AD. Andrews et al. (2018) unfolded 12 single nucleotide poly morphisms significantly associated with baseline cognitive performance, linear rate of change, or quadratic rate of change. In the age-related gene segments, APOE e4 allele was linked to poor cognitive aging and enhanced dementia risk (Evans et al., 2017). Such an influence of APOE e4 on cognitive decline with age was observed earlier in lifespan, ranging from about 30 years old (Evans et al., 2017) to mid-adulthood (before about 60 years old; Lancaster et al., 2017). Nevertheless, Rantalainen et al. (2016) observed that APOE major isoforms was not associated with cognitive ability but the number of minor alleles in rs405509 and rs440446 polymorphisms was relevant to higher cognitive ability in older adults independent of the APOE major isoforms.

**Figure 5:**
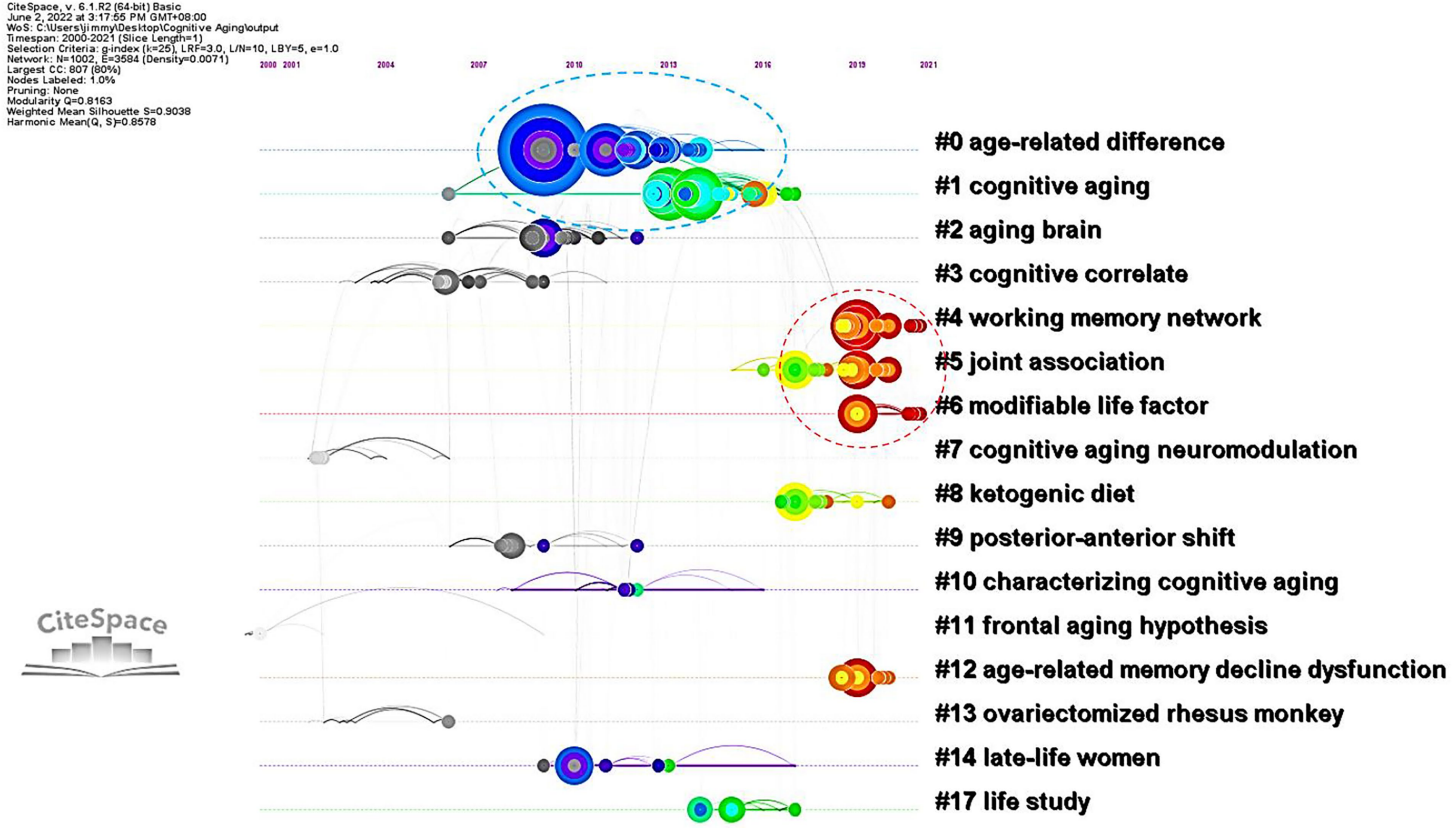
A timeline visualization of the co-citation network in CNA.

The six groups of research mentioned above were synchronically delineated based on their frequently cited references in each cluster of Figure 4. Nevertheless, Figure 4 did not display an epochal character of CNA research because each group contained many colorful circles (especially in the middle of Figure 4) and only the left and right sides showed black and red colors, respectively. Therefore, we presented Figure 5, which showed a timeline visualization of the co-citation network in CNA research.

The timeline visualization explicitly delineated the development of each cluster in Figure 4 and displays the information flow among them. Although Figure 4 showed a mixture of colorful circles, the timeline visualization in Figure 5 manifested two groups of serried circles from 2008 to 2014 and from 2016 to 2021. The first period covered the first two clusters, and the second period contains clusters #4, #5, and #6. The figure informed us that the discussion of CNA had once attracted a lot of interests from scholars in the period 2008–2014, and studies increasingly started to concentrate on the cognitive mechanism of CNA or the factors influencing aging or influenced by aging. However, the links (i.e., grey filament between clusters) were not distributed intensively or convergingly and other clusters did not have many circles. This meant that each cluster in CNA might have its own trajectory, and they were not connected closely as suggested by only a few information flows among them.

To reveal the influential works in CNA, we listed both the top 10 references in terms of citation counts and the top 10 in terms of BC values and examined what they have discovered for the development of CNA research based on co-citations (see Table 3). Although Figures 3, 4 listed 16 clusters of CNA research, the influential references only occurred in half of them (i.e., clusters #0 – #3, #11, and #12). These references focused on the theories and neuromechanism of CNA, which existed as the basis of the studies of factors and diseases concerning CNA. For the top 10 references in terms of frequency, the main focus was “(working) memory” during the brain aging process, with investigations from the perspective of brain maintenance (Nyberg et al., 2012) and brain changes (Park et al., 2002; Grady, 2012; Cabeza et al., 2018). Regarding the studies on brain changes, scholars particularly focused on cognitive reserve and compensation. Meanwhile, references with high BC values also covered the studies of functional or volume changes of different brain areas in CNA except for those that focused on the factor of memory. Those brain areas included the medial frontal cortex relating to cognitive performance (Davis et al., 2008), hippocampus relating to scene encoding (Gutchess et al., 2005; Park and Reuter-Lorenz, 2009), the compensation neural networks in the brain (prefrontal cortex, medial temporal lobes, fusiform face area, parahippocampus area, posterior cingulate cortex, and the like; Grady, 2012), and the shrinkage of the cerebellum, hippocampus, entorhinal cortices, inferior temporal cortex, and prefrontal white matter (Raz et al., 2005).

**Table 3:** References with top 10 frequency and BC value.

Freq.	Author	Title	Source	Cluster
35	Park & Reuter-Lorenz	The Adaptive Brain: Aging and Neurocognitive Scaffolding	*Annual Review of Psychology*	#0
21	Stern	Cognitive reserve	*Neuropsychologia*	#0
18	Nyberg et al.	Memory aging and brain maintenance	*Cell*	#1
17	Cabeza et al.	Maintenance, reserve and compensation: the cognitive neuroscience of healthy ageing	*Nature Reviews*	#4
17	Davis et al.	Qué PASA? The Posterior--Anterior Shift in Aging	*Cerebral Cortex*	#2
16	Livingston, et al.	Dementia prevention, intervention, and care	*The Lancet Commissions*	#12
16	Grady	The cognitive neuroscience of ageing	*Nature Reviews*	#1
15	Stern et al.	Whitepaper: Defining and investigating cognitive reserve, brain reserve, and brain maintenance	*Alzheimer’s & Dementia*	#4
14	Salthouse	When does age-related cognitive decline begin?	*Neurobiology of Aging*	#14
14	Stern	Cognitive reserve in ageing and Alzheimer’s disease	*Lancet Neurology*	#14
BC	Author	Title	Source	Cluster
0.36	Park & Reuter-Lorenz	The Adaptive Brain: Aging and Neurocognitive Scaffolding	*Annual Review of Psychology*	#0
0.36	Grady	The cognitive neuroscience of ageing	*Nature Reviews*	#1
0.23	Gutchess et al.	Aging and the Neural Correlates of Successful Picture Encoding: Frontal Activations Compensate for Decreased Medial-Temporal Activity	*Journal of Cognitive Neuroscience*	#2
0.20	Chan et al.	Decreased segregation of brain systems across the healthy adult lifespan	*PNAS*	#1
0.17	Davis et al.	Qué PASA? The Posterior--Anterior Shift in Aging	*Cerebral Cortex*	#2
0.13	Nyberg et al.	Memory aging and brain maintenance	*Cell*	#1
0.13	Raz et al.	Regional Brain Changes in Aging Healthy Adults: General Trends, Individual Differences and Modifiers	*Cerebral Cortex*	#3
0.11	Bennett & Madden	Disconnected aging: cerebral white matter integrity and age-related differences in cognition	*Neuroscience*	#1
0.10	Baddeley	Working memory	*Current Biology*	#11
0.09	Cabeza	Hemispheric Asymmetry Reduction in Older Adults: The HAROLD Model	*Psychology and Aging*	#7

Aside from the topics of memory and changes of aging brain areas, the references presented in Table 3 also examined CNA theories and its neuromechanism. These references evidently revealed the influential status of the scaffolding theory (Park and Reuter-Lorenz, 2009), the hemispheric asymmetry reduction in older adults (HAROLD) model (Cabeza, 2002), and the posterior–anterior shift (Davis et al., 2008).

### 3.2. Co-word analysis (I): Detail information for complementing the co-citation analysis

The co-citation analysis revealed some major fields in CNA studies and presented some refined topics such as brain area (or location), theory, or neural mechanism. Considering that the information was obtained from only highly cited references (or articles) in Table 3 and Figure 4, the major fields of CNA required more evidence for additional support. Therefore, we carried out a co-word analysis to refine the keywords from the data collected by CiteSpace, and we also extracted the keywords with high frequency and BC values in each decade, exhibited in Table 4.

**Table 4:** Keywords with high frequency and BC (> 0.1) value.

2000 ~ 2010 (TOP 20)					
BC	Freq.	Keywords	Freq.	BC	Keywords
0.33	20	age	65	0.17	cognitive aging
0.31	13	cognitive decline	52	0.29	alzheimers disease
0.29	52	alzheimers disease	33	0.11	older adult
0.23	20	age related change	29	0.03	working memory
0.20	7	apolipoprotein e	26	0.04	performance
0.19	12	individual difference	23	0.03	memory
0.17	65	cognitive aging	20	0.33	age
0.16	16	spatial memory	20	0.23	age related change
0.16	16	brain	20	0.10	prefrontal cortex
0.16	13	decline	16	0.16	spatial memory
0.16	12	adult age difference	16	0.16	brain
0.15	9	attention	13	0.11	dementia
0.15	7	cognitive function	13	0.05	recognition memory
0.13	4	cerebral cortex	13	0.16	decline
0.12	5	activation	13	0.31	cognitive decline
0.12	3	central nervous system	12	0.16	adult age difference
0.11	33	older adult	12	0	risk
0.11	13	dementia	12	0.19	individual difference
0.11	11	executive function	12	0.01	brain activity
0.11	4	beta amyloid accumulation	11	0.11	executive function
2011 ~ 2021 (TOP 13)					
**BC**	**Freq.**	**Keywords**	**Freq.**	**BC**	**Keywords**
0.22	9	parkinsons disease	392	0	cognitive aging
0.21	8	hippocampal	317	0	alzheimers disease
0.19	18	MRI	169	0.01	older adult
0.16	17	gray matter	134	0.04	dementia
0.14	27	human brain	115	0.02	working memory
0.13	68	prefrontal cortex	113	0.03	impairment
0.12	34	physical activity	106	0.01	memory
0.12	16	dentate gyrus	106	0.02	mild cognitive impairment
0.12	13	cohort study	102	0.02	age
0.11	56	cognitive decline	95	0.02	decline
0.11	31	age di fference	92	0.01	performance
0.11	22	attention	88	0.02	executive function
0.11	5	aerobic exercise	82	0.10	episodic memory

We first listed the keywords with high BC values (over 0.1) in the two decades and then listed an equal number of keywords with high frequency. Intuitively, the keywords with high BC values in the first decade were more than those in the second decade. Compared with the co-citation analysis, the co-word analysis provided more detailed information in terms of the impacting factors, brain areas, and aging diseases in CNA research. In Table 4, “(working) memory” was a popular topic with high frequency. However, the co-word analysis showed most subtypes of memory such as spatial memory, recognition memory, and episodic memory. Moreover, “attention” occurred throughout the two decades with a high BC value, implying that this topic attracted long-term attention in CNA research. In the investigation of memory and attention of older adults, the brain or cognitive changes in executive functions, cognitive decline, individual differences (e.g., age or sex), and cognitive impairment also became important keywords across the two decades. These results were consistent with those of cluster analysis.

### 3.3. Co-word analysis (II): Age-related brain areas

As for the brain area, the co-word analysis did not show anything unusual in comparison with those in the co-citation analysis. The hippocampus, prefrontal cortex, grey matter, dentate gyrus, and central nervous system emerged with high BC values, which suggested that future transformative discoveries might be found in these areas. Except for the hot topics and brain areas, the co-word analysis further revealed two major diseases in CNA research, as expected, namely, AD (or senile dementia) and Parkinson’s disease (PD), in both decades. Nevertheless, the study of PD had an extremely high BC value especially during 2011 to 2021, and AD did not have a BC value over 0.1 in the same period. This result might indicate a tendency of the flow of interests from the investigation of AD to PD. The data in Table 4 also showed some crucial biomarkers (e.g., apolipoprotein E and β amyloid accumulation) concerning the two diseases, and an intervention method (i.e., aerobic exercise) to postpone the occurrence of AD and PD.

**Figure 6:**
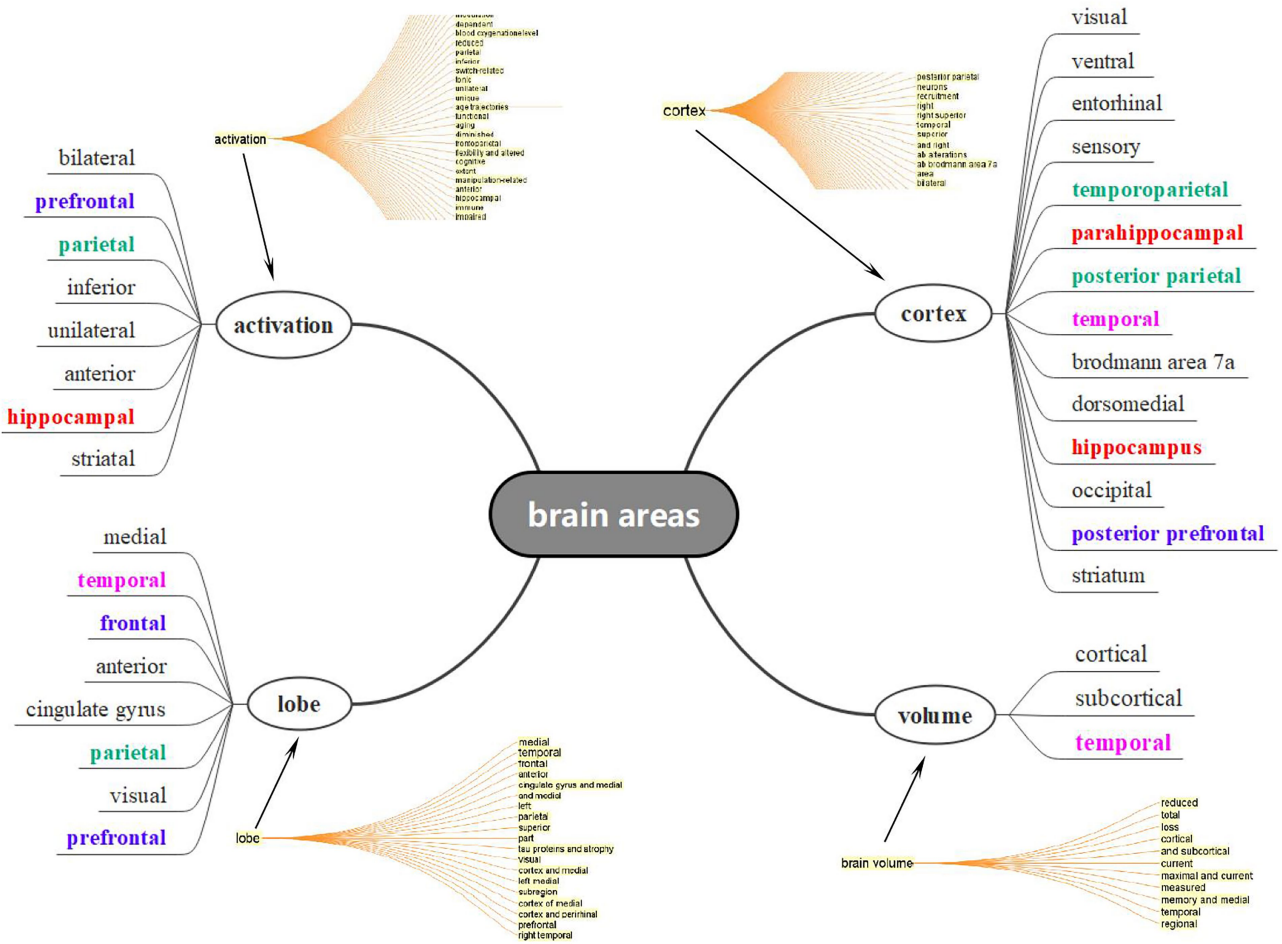
Crucial brain areas in CAN based on concept tree results in Cite Space.

Considering the facts that there were numerous areas and locations concerning the aging brain, ranging from the prefrontal to occipital regions and from grey matter to dentate gyrus, it was impossible for us to list all the areas concerning the aging process. Under this circumstance, we therefore extracted the title and abstract from the data, generalized a concept tree in CiteSpace, and selected all expressions concerning brain areas that lay within the four bigger concepts in Figure 6, based on the syntactic structure and dependency parsing function of CiteSpace. As expected, more than 30 brain areas were observed in CNA studies and each of them might be interpreted from the perspectives of volume, location (or distribution), and function. Nevertheless, some areas in different colors still occurred frequently in Figure 6. An important concept presented in Figure 6 was at the bottom right corner, namely, “volume” whose items were very small but general. This indicated that the volume changes regarding aging brains cover all the brain areas, as introduced by Nyberg et al. (2012), from early adulthood. Owing to the shrinkage of the brain, serious changes in cognitive functions and compensations occurred in the aging brains. Three major areas (or lobes) appeared to be more important than others in CNA, namely, the temporal (especially the hippocampus), prefrontal, and parietal lobes.

## 4. Discussion

Generally, the 20-year development of CNA manifested a growing tendency for its publication, and four aspects have been explored during the past two decades, namely, factor, theory, brain area (including neuromechanism), and diseases. This section will canvass in detail the three research questions based on these four aspects.

### 4.1. Important and popular research topics and development stage of CNA studies

Both the co-citation and co-word analyses has revealed that “memory” and “attention” are the most popular topics (or research objectives) in CNA, not only because these two topics are important in psychology and neuroscience but also because they relate to the cognitive decline people experience as they get older, and to the two primary CNA diseases (i.e., AD and PD). The proportion of the elderly population is gradually increasing internationally as human life is prolonged by improvements in medical treatments and the standards of living (Boland and Mark, 2012). Meanwhile, neurodegenerative diseases have become more prevalent from the beginning of this century and may become extremely serious, threatening human health (Ou et al., 2021). More importantly, no highly effective drugs or therapies currently exist to prevent these neurodegenerative diseases, even though the medical research on them has already advanced rapidly (Hogan, 2014; Bateman, 2015). Such a situation brings great pressure and challenges for medical workers, patients’ families, and governments. Therefore, AD and PD inevitably became the center of CNA studies, and the popular argumentations are spontaneously driven by the studies of these two diseases.

AD and PD are totally different diseases, even though they both show retrogressive processes in older adults’ brain. Nevertheless, they show some similarities during their advanced stages. For AD, anosognosia (a deficit in self-awareness) is one of the core symptoms as it deteriorates to an advanced stage (Maki et al., 2016), and anosognosia is typically manifested in cognitive domains, especially in memory function (Souchay, 2007). As for PD, although primary lesion may cause a disorder in old adults’ behavior (e.g., tremor or bradykinesia), its further development can lead to diverse symptoms such as poor memory, anxiety, apathy, or depression (Forsyth and Meara, 2016). As for the investigations of attention in CNA, an internationally agreed-upon criterion for the diagnosis of dementia with Lewy bodies is that memory impairment may not necessarily occur in the early stage of PD dementia, but there exist prominent attentional deficits (Hindle, 2016). These concepts justify the status of memory and attention in CNA; thus, they naturally become the two most influential topics that present transformative discoveries in future studies.

Although the studies in the field of CAN has produced several valuable findings about memory and attention in AD and PD, the results of the co-citation and co-word analyses still suggest that it is at the early stage, not only because such a discipline is younger than other neuroscientific branches but also because the influential articles (especially the review articles) converge in presenting the newly defined targets like those influential references introduced in the 16 clusters, which, according to Shneider (2009), is characteristic of the first stage of the evolution of a scientific discipline. In Shneider (2009) theory, the evolution of a scientific discipline is divided into four stages. In the first stage, scholars are busy with the new subject matter in the realm of scientific analysis to introduce a system of their own jargons that adequately describe new findings in a particular topic such as “STAC model,” “neuroplasticity,” “functional compensation,” “posterior–anterior shift,” and “APOE e4” in those 16 clusters. All these expressions are established to describe particular results in the study of CNA. Another point suggesting that the development of CNA is at its first stage (i.e., a primary stage) in which “scientists rarely focus their interests on one single field of study” (Shneider, 2009, p: 219). The results of co-citation and co-word analyses verify this point regarding the 20-year development of CNA. They contain four areas realized by 16 clusters, with only approximately one thousand articles—ranging from human brain changes and disease intervention to zoopery.

Although the present published articles display a primary stage of CNA, the occurrence of its second stage is predicted in our results. As explained by Shneider (2009), the second stage of a scientific field is characterized by the development of research instrument or equipment that enable scholars to probe into hidden phenomena. Such a tendency has already revealed in the co-word analysis. For instance, the (f) MRI occurs in Table 4 in the period 2011–2021 with a relatively high BC value, indicating possible transformative discoveries in the future (Chen et al., 2009; Chen, 2017). This is consistent with Shneider’s four-stage theory. Nevertheless, other neuroscientific techniques such as ERP, DTI, PET, or MEG do not show up in the co-word analysis results. The fMRI-oriented tendency can also be identified with the number of publications in the WOS core collection. The summation of the published articles concerning CNA by the non-(f) MRI studies is surprisingly less than those by (f)MRI. Consequently, the second stage of CNA may also at a departure point, and more equipment may be employed in the future, which would probably stimulate the creation of novel research that employs other equipment and enables CNA to enter its third stage (i.e., large-scale application stage) of development.

Moreover, in the first stage of development, CNA studies have displayed an imbalance between “memory and attention” vs. “other cognitive process” (e.g., language, emotion, perception, etc.) The increasing studies on memory and attention in CNA is due to the increasing attention to AD and PD, and other phenomena like language and emotion indeed play significant roles in the progress of the two diseases as mentioned previously. That is, the advanced stage of both diseases covers the symptoms of language disorder and abnormal emotions (e.g., apathy, depression, or anxiety; Souchay, 2007; Forsyth and Meara, 2016; Maki et al., 2016). In the data we collected, only 63 articles concern language, and 58 articles concern the emotions of old adults in the past two decades. This unbalanced development may have resulted from the different status of memory, attention, language, and emotion in the two diseases. Apparently, memory and attention are at the center of CNA studies, while language and emotion may be influenced by the decline in memory and attention.

### 4.2. The influential theories in the domain of CNA

Two influential theories are revealed in our results, namely, the STAC model and the HAROLD model. The STAC was proposed by Park and Reuter-Lorenz (2009); it integrates extant cognitive, functional, and structural imaging data, leading to predictions about the brain’s plasticity in changing scenarios. It explains how the combination of compensatory and adverse neural processes realizes various levels of cognitive function (Reuter-Lorenz and Park, 2014). The scaffolding is defined as “the recruitment of additional circuitry that shores up declining structures whose function has become noisy, inefficient, or both” (Park and Reuter-Lorenz, 2009, p: 183). The definition hints at the existence of a continuous engagement of compensatory scaffolding in our brain, which is characterized by Park and Reuter-Lorenz (2009) as neurally dynamic and an ongoing property of an adaptive brain across one’s lifespan. The uniqueness of STAC in older adults is that compensatory scaffolding may occur under conditions of less novel or practiced behaviors because of the degraded neural circuitry for performing specific tasks (Goh and Park, 2009).

The significant status of STAC may have resulted from two aspects. The first one is that STAC is supported by evidence from behavioral theories on aging, structural brain changes, neurobiological changes in the aging brains, and functional imaging of the aging brain (Goh and Park, 2009). The behaviorally aging performance is relevant to the decline and presentation in terms of verbal knowledge (Hultsch et al., 1998; Park et al., 2002) and executive processing functions such as working memory and attentional processing (Goh and Park, 2009). The structural changes in aging brains are evidenced by the shrinkage of brain volume, cortical thickness, and white-matter integrity (Salat et al., 2004; Wen and Sachdev, 2004; Raz et al., 2005). Neurobiological evidence from the dysregulation of dopamine receptors in the frontal regions explains the observed behavioral decline in old adults’ ill performance (Kaasinen et al., 2000). In addition, the functional imaging literature provides distinct proof of greater breadth of activation in old adults’ brain than in young adults when performing a cognitive task, especially in the frontal cortex. Another perspective for understanding the influential status of STAC is that it deems the aging process as not only dynamic but more importantly, adaptive. It delineates how the aging brain confronts the challenges from neurodegenerative diseases or other physical or medical interventions that will reduce the cognitive ability.

In the functional imaging evidence for STAC, the HAROLD model by Cabeza (2002) is the second most influential theory in the field of CAN; HAROLD was introduced earlier than the STAC. This model states that “under similar circumstances, prefrontal activity during cognitive performances tends to be less lateralized in older adults than in younger adults” (Cabeza, 2002, p: 85), and it is supported by evidence from studies on episodic memory (Tulving, 1983; Nyberg et al., 1996, 1998), semantic memory (Logan and Buckner, 2001; Stebbins et al., 2002), working memory (Dixit et al., 2000; Reuter-Lorenz et al., 2000), perception (Grady et al., 1994, 2000), and inhibition (Garavan et al., 1999; Nielson et al., 2002). Cabeza (2002) has tried to interpret the HAROLD model in terms of its function, from compensation vs. dedifferentiation views; its origin, from psychological vs. neurogenic views; and the neural mechanisms, from network vs. regional views; and justified that such a model is consistent with other cognitive aging theories (e.g., resources, speed, and inhibition theories). However, this model concerns only the effect of aging in the prefrontal cortex rather than other areas. This may be the reason the article concerning the HAROLD model is assigned a relatively low BC as shown in Table 3 compared with the STAC, which is assigned the top rank in both frequency and BC value and considered the most possible area to produce transformative findings in the future.

From the perspective of bibliometric analysis, the development of these two theories reflects an information flow from CA to CNA. To testify the two theories, scholars conducted abundant research in an attempt to find evidence to support the STAC and HAROLD models. The references listed in the above tables covered a wide range of subareas—from cognitive psychology to the neuroscience of aging. As Cabeza (2001) stated that the *cognitive psychology of aging* and the *neuroscience of aging* are two well-established disciplines; thus, employing the research findings from these two disciplines as evidence makes the two theories convincing and reliable. Furthermore, the combination of the two disciplines may also indicate that there are some issues about CA that cannot be answered only through cognitive psychology, which finally stimulates the occurrence of CNA.

### 4.3. Age-related changes in different brain areas

The first crucial brain area relating the memory of old adults, including the hippocampus and the frontal lobe. The hippocampus (sometimes called the medial temporal lobe) seems to be the center of memories in the brain (Hayes and Stratton, 2022). This key status of the hippocampus is consistent with the hot topics revealed by the co-citation analysis—that memory possesses a crucial status in CNA research. The prefrontal cortex (PFC) is an area on the frontal lobes, in front of the premotor cortex; it is responsible for planning and higher-level control or regulation of actions (Hayes and Stratton, 2022). According to the references in clusters #0 and #2, the hippocampus and PFC are vulnerable to age-related changes in structure and activities of the human brain (Jackson et al., 2009; Wang and Michaelis, 2010). A decline or impairment of the PFC would influence old adults’ executive function, including motivation, sustained attention, planning, working memory, and sequencing (Barter and Foster, 2020). Meanwhile, the hippocampus is closely related to the decline in episodic memory among older adults or even middle-aged people (Cansino, 2009; Nyberg et al., 2012). Aside from the shrinkage of the hippocampus that causes the decline in cognitive execution, Nelson et al. (2019) and Brenowitz et al. (2014) proposed that people who suffer from hippocampal sclerosis of aging usually show a severe episodic memory defect, just like the symptom of AD.

The second important brain area, namely the parietal lobe, plays an important role of intersection in functional compensation. The parietal lobe is a large area of the cerebrum behind the central fissure and above the occipital lobe (Hayes and Stratton, 2022). This area is responsible for integrating visual and somatosensory information (e.g., temperature and pain; Clarke, 2022). It is a highly complicated location because it connects with many processes and other areas to construct networks. A group of studies have suggested that the posterior parietal area is relevant to executive control (e.g., attentional switching; Hampshire et al., 2008); prefrontal−/frontal–parietal, to working memory (Hakun and Johnson, 2017; Heinzel et al., 2017); frontal–parietal network, to a demand for a cognitive control (Cohen et al., 2020); and inferior parietal, to attentional demands (Kim and Giovanello, 2011). Although abundant studies have provided numerous evidence regarding the function of the parietal area in CNA, and it is difficult to list all of them, one significant clue can be found from the given areas in Figure 6 and Tables 2, 3. That is, the connection between the anterior and posterior areas is a very valuable point, especially for the investigations of brain areas in CNA. Other research took the PFC and parietal areas as a dorsal attention network (Corbetta et al., 2008; Vincent et al., 2008), displaying more activity in older than in younger adults. This finding informs that the parietal region is highly active in constructing neural networks and may be a critical intersection in fulfilling the functional compensation.

As for the third important brain area, temporal lobe—an area of the cerebrum below the lateral fissure and at the side of each cerebral hemisphere (Hayes and Stratton, 2022)—like the parietal area, is also a complicated area that is relevant to aphasia, prosopagnosia, aggression, and amnesia (Clarke, 2022). The temporal area is discussed repeatedly in terms of its relation to the PFC, hippocampus, and the parietal area. More importantly, the discussion on the relationship between the temporal and other areas also concerns the compensatory relationship between the PFC and the temporal lobe, which is evidenced by less activation in the left and right temporal lobes (more precisely, the parahippocampus) of old adults compared with young adults, and more activation in the middle frontal area in old adults than in young adults (Gutchess et al., 2005).

**Figure 7:**
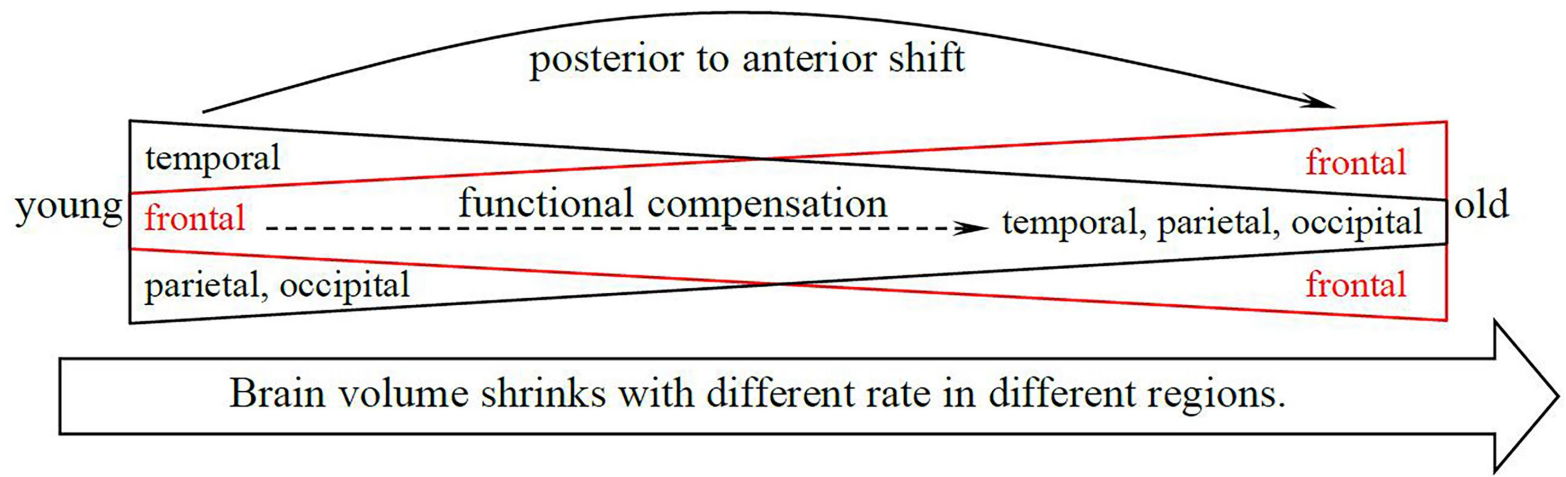
The compensatory relation between the brain areas in the studies of topics relation to memory and attention in old adults.

In general, the co-word analysis presents four primary brain areas/regions, and the co-citation analysis results provide further information regarding the functions and functionally compensatory relationship between them. First, studies on the functions and volumes of different brain areas in CNA research agree that the human brain starts to shrink from early middle age but with different rates in different regions. Second, the three major brain areas are all relevant to memory and attention resources, which makes the hippocampus and PFC highly significant in CNA. Finally, most of the CNA studies with high frequency and BC values concern the compensatory relation between the posterior and anterior regions and mention a hypothesized functionally compensatory approach from the frontal to the temporal, parietal, and occipital areas. We generalize such a compensatory relation between different areas in old adults as shown in Figure 7. Compared with younger adults’ frontal lobe, the older adults’ frontal lobe is always more activated while less activated in temporal, parietal, and occipital lobes as the brain shrinks during one’s lifespan. These findings align with the findings of the main research objectives and theories in CNA. Owing to the concentration on memory and attention, the temporal (especially the hippocampus) and frontal lobes are two inevitable areas in the CNA field. Moreover, the compensatory relations between the posterior and the anterior regions further sustained the dynamic changes and adaptive characteristics of the human brain through a lifespan delineated by the scaffolding theory and the HAROLD model.

It should be noted that only the occipital lobe did not show up in the results of co-word analysis. This only indicated that this area has so far not been examined frequently and did not mean that it is unimportant in CNA research. Occipital lobe is responsible for visual processing, especially for faces, but it is not responsible for processing facial identity (Hayes and Stratton, 2022). In this case, one plausible reason is that lower-level visual processing is not frequently examined during this period.

## 5. Conclusion

This study focused on the development of CNA to identify the highly influential and potential research topics, theories, and (age-related changes) brain area based on a bibliometric analysis *via* CiteSpace. The co-citation and co-word analyses revealed that although CNA research put priority on concentrated on AD and PD, “memory” and “attention” of old adults were the two main focuses in this discipline. Scholars of CNA were interested in the decline in memory and attention in AD and PD patients. Meanwhile, the studies on memory and attention were gradually progressing toward an experimental equipment-oriented stage, but there is still space for neuroscience techniques to develop. Based on these experimental results, a series of views, concepts, and theories have been proposed, among which the scaffolding theory and HAROLD model were the two influential theories which would probably lead to transformative discoveries. The competitive point of the theories was that they treat the decline in people’s cognitive abilities as a dynamic and compensatory process rather than a simple or absolute cognitive decline. Such a point has been identified through the volume shrinkage rate in different brain areas and the fMRI studies concerning the cognitive aging changes in the functions of temporal (especially the hippocampus), frontal, and parietal lobes, which presented a compensatory relationship between the anterior and posterior regions.

There were also some less investigated topics such as the language and emotion processing of older adults or of the cognitive diseases of patients, which have not been identified as popular topics in the results. The impairment in the mentioned brain areas is relevant to language and emotion, but the research on these two phenomena is much less than that on memory and attention. Another topic that should be considered in the future is the relation between factors (e.g., vascular disease, genic disease, and environment) that cause CNA diseases and the compensatory relations, because a recent study suggested that dementia is influenced differently by the vascular risk factors (Emer et al., 2022). Therefore, research gap like whether the decline in cognitive functions caused by hippocampus sclerosis and by atrial fibrillation share the same functional compensation in the neural network of aging brains is a topic that deserves further study.

## Author contributions

JJ is responsible for the draft manuscript preparation, revision, and the data analysis. LF is responsible for the conception, design, draft manuscript preparation, revision and giving suggestions for the interpretation of the results. JL is responsible for the figures, tables, and revision. All authors reviewed the results and approved the final version of the manuscript.

## Funding

This work was supported by research grants awarded by National Planning Office of Philosophy and Social Science [18BYY088].

## Conflict of interest

The authors declare that the research was conducted in the absence of any commercial or financial relationships that could be construed as a potential conflict of interest.

## Publisher’s note

All claims expressed in this article are solely those of the authors and do not necessarily represent those of their affiliated organizations, or those of the publisher, the editors and the reviewers. Any product that may be evaluated in this article, or claim that may be made by its manufacturer, is not guaranteed or endorsed by the publisher.

## Figures and Tables

**Table:** 

Freq.	Author	Title	Source	Cluster
31	Reuter-Lorenz and Park, (2014)	How does it STAC Up? Revisiting the scaffolding theory of aging and cognition	*Neuropsychology Review*	#0
19	Meusel et al. (2014)	A systematic review of type 2 diabetes mellitus and hypertension in imaging studies of cognitive aging: time to establish new norms	*Frontiers in Aging Neuroscience*	
19	Raz and Rodrigue (2006)	Differential aging of the brain: Patterns, cognitive correlates and modifiers	*Neuroscience and Biobehavioral Reviews*	#3
17	Li and Sikström (2002)	Integrative neurocomputational perspectives on cognitive aging, neuromodulation, and representation	*Neuroscience and Biobehavioral Reviews*	#7
17	Greenwood (2000)	The frontal aging hypothesis evaluated	*Journal of the International Neuropsychological Society*	#11
16	Krivanek et al. (2021)	Promoting successful cognitive aging: A ten-year update	*Journal of Alzheimer’s Disease*	#6
16	Li and Lindenberger (2002)	Relations between aging sensory/sensorimotor and cognitive functions	*Neuroscience and Biobehavioral Reviews*	#7
14	Goh and Park (2009)	Neuroplasticity and cognitive aging: The scaffolding theory of aging and cognition	*Restorative Neurology and Neuroscience*	#2
13	Greenwook and Parasuraman (2010)	Neuronal and cognitive plasticity: A neurocognitive framework for ameliorating cognitive aging	*Frontiers in Aging Neuroscience*	#2
13	Reinvang et al. (2010)	Neurogenetic effects on cognition in aging brains: A window of opportunity for intervention?	*Frontiers in Aging Neuroscience*	
13	Fjell et al. (2009)	High consistency of regional cortical thinning in aging across multiple samples	*Cerebral Cortex*	#3
13	Abellaneda-Pérez et al. (2019)	Combining non-invasive brain stimulation with functional magnetic resonance imaging to investigate the neural substrates of cognitive aging	*Journal of Neuroscience Research*	#4
12	Eyler et al. (2011)	A review of functional brain imaging correlates of successful cognitive aging	*Biological Psychiatry*	#2
12	Greenwood (2007)	Functional Plasticity in Cognitive Aging: Review and Hypothesis	*Neuropsychology*	#3
12	Chee et al. (2009)	Cognitive function and brain structure correlations in healthy elderly East Asians	*NeuroImage*	
12	Alexander et al. (2012)	Characterizing cognitive aging in humans with links to animal models	*Frontiers in Aging Neuroscience*	#10
11	Anstey et al. (2007)	Corpus callosum size, reaction time speed and variability in mild cognitive disorders and in a normative sample	*Neuropsychologia*	#3
11	Pettigrew and Soldan (2019)	Defining Cognitive Reserve and Implications for Cognitive Aging	*Current Neurology and Neuroscience Reports*	#4
11	Davis et al. (2008)	Qué PASA? The Posterior–Anterior Shift in Aging	*Cerebral Cortex*	#9
11	Rapp et al. (2003)	Cyclic estrogen replacement improves cognitive function in aged ovariectomized rhesus monkeys	*The Journal of Neuroscience*	#13
10	Gauthier et al. (2013)	Age dependence of hemodynamic response characteristics in human functional magnetic resonance imaging	*Neurobiology of Aging*	#0
10	Bennett and Madden (2013)	Disconnected aging: cerebral white matter integrity and age-related differences in cognition	*Neuroscience*	
10	Siman-Tov et al. (2017)	Early age-related functional connectivity decline in high-order cognitive networks	*Frontiers in Aging Neuroscience*	#1
10	Daffner (2010)	Promoting Successful Cognitive Aging: A Comprehensive Review	*Journal of Alzheimer’s Disease*	#2
10	Madden et al. (2009)	Cerebral white matter integrity and cognitive aging: Contributions from diffusion tensor imaging	*Neuropsychology Review*	
10	Duggan and Parikh (2021)	Microglia and modifiable life factors: Potential contributions to cognitive resilience in aging	*Behavioral Brain Research*	#6
10	Cabeza et al. (2004)	Task-independent and task-specific age effects on brain activity during working memory, visual attention and episodic retrieval	*Cerebral Cortex*	#7
10	Hernandez et al. (2018)	A ketogenic diet improves cognition and has biochemical effects in prefrontal cortex that are dissociable from hippocampus	*Frontiers in Aging Neuroscience*	#8
10	lacreuse (2006)	Effects of ovarian hormones on cognitive function in nonhuman primates	*Neuroscience*	#13

## Footnotes

^1^The ESCI started from 2015 and articles on CNA started to occur in 2017. Therefore, the data collected from ESCI is from 2017 to 2021.

^2^According to Cabeza et al. (2017, p: 1), "since 2004, cognitive neuroscience of aging has continued to expand at a very rapid pace…it is fair to say that the number of articles in cognitive neuroscience of aging published per year has tripled during the last 10 years." It meant that CNA had gradually form its theoretical ground, popular research fields, and methodologies in the past two decades. Considering the research purposes and questions, therefore, we selected the time span from 2000 to 2021.

^3^The PRISM 2020 statement for systemic review can be found in Page et al. (2021).

^4^The augmentation of the publication of CNA may also be influenced by the occurrence of ESCI after 2015. However, there is no citation burst after 2015, and thus we do not consider the occurrence of ESCI as the main reason of the increase in publication counts after 2015.

^5^These groups apparently cover numerous references, and our depiction can only introduce some of them relating to the major topic or argumentation in each cluster. Under this circumstance, we listed the influential papers (citation counts ≥10) in each cluster in the Appendix for readers’ reference.
